# Management of perimesencephalic nonaneurysmal subarachnoid hemorrhage: a national survey

**DOI:** 10.1038/s41598-023-39195-2

**Published:** 2023-08-07

**Authors:** Christina Wolfert, Christoph J. Maurer, Björn Sommer, Kathrin Steininger, Stefan Motov, Maximilian-Niklas Bonk, Philipp Krauss, Ansgar Berlis, Ehab Shiban

**Affiliations:** 1 Department of Neurosurgery, University Hospital Augsburg, Stenglinstr. 2, 86156 Augsburg, Germany; 2 Department of Neuroradiology, University Hospital Augsburg, Stenglinstr. 2, 86156 Augsburg, Germany

**Keywords:** Neurological disorders, Cerebrovascular disorders

## Abstract

Perimesencephalic nonaneurysmal subarachnoid hemorrhage (NASAH) is a rare type of subarachnoid hemorrhage (SAH), usually associated with minor complications compared to aneurysmal SAH. Up to date, data is scarce and consensus on therapeutic management and follow-up diagnostics of NASAH is often missing. This survey aims to evaluate the clinical management among neurosurgical departments in Germany. 135 neurosurgical departments in Germany received a hardcopy questionnaire. Encompassing three case vignettes with minor, moderate and severe NASAH on CT-scans and questions including the in-hospital treatment with initial observation, blood pressure (BP) management, cerebral vasospasm (CV) prophylaxis and the need for digital subtraction angiography (DSA). 80 departments (59.2%) answered the questionnaire. Whereof, centers with a higher caseload state an elevated complication rate (Chi^2^ < 0.001). Initial observation on the intensive care unit is performed in 51.3%; 47.5%, 70.0% in minor, moderate and severe NASAH, respectively. Invasive BP monitoring is performed more often in severe NASAH (52.5%, 55.0%, 71.3% minor, moderate, severe). CV prophylaxis and transcranial doppler ultrasound (TCD) are performed in 41.3%, 45.0%, 63.8% in minor, moderate and severe NASAH, respectively. Indication for a second DSA is set in the majority of centers, whereas after two negative ones, a third DSA is less often indicated (2nd: 66.2%, 72.5%, 86.2%; 3rd: 3.8%, 3.8%, 13.8% minor, moderate, severe). This study confirms the influence of bleeding severity on treatment and follow-up of NASAH patients. Additionally, the existing inconsistency of treatment pathways throughout Germany is highlighted. Therefore, we suggest to conceive new treatment guidelines including this finding.

## Introduction

In 2013 the European Stroke Organization published treatment guidelines for SAH, thus mainly concentrating on aneurysmal SAH, also the treatment of NASAH was integrated^1^. Differentiating SAH into perimesencephalic and non-perimesencephalic distribution determines the therapeutic approach^2^. Since perimesencephalic NASAH is associated with minor complication rates regarding rebleeding, CV, and delayed cerebral ischemia (DCI) the European Stroke Organization does not recommend repeated digital subtraction angiography (DSA) in these cases^1,3,4^. Balancing the periprocedural risk against the chance of a pathological finding in repeated DSA^1,5^. However, the recommendations of this guideline are limited due to the lack of prospective data. Therefore, our survey includes three case vignettes encompassing minor, moderate and severe perimesencephalic NASAH to represent real-world data from neurosurgical departments nationwide. The aim of this study is to provide information on current management of NASAH in Germany and indicate suggestions of improvement in patient care.

## Materials and methods

### Study setting

Paper-based questionnaires were sent to all neurosurgical departments in Germany. Including questions to outline the case load and the estimated complication rate, mainly focusing on CV. For more information, see supplementary file.

**Figure 1:**
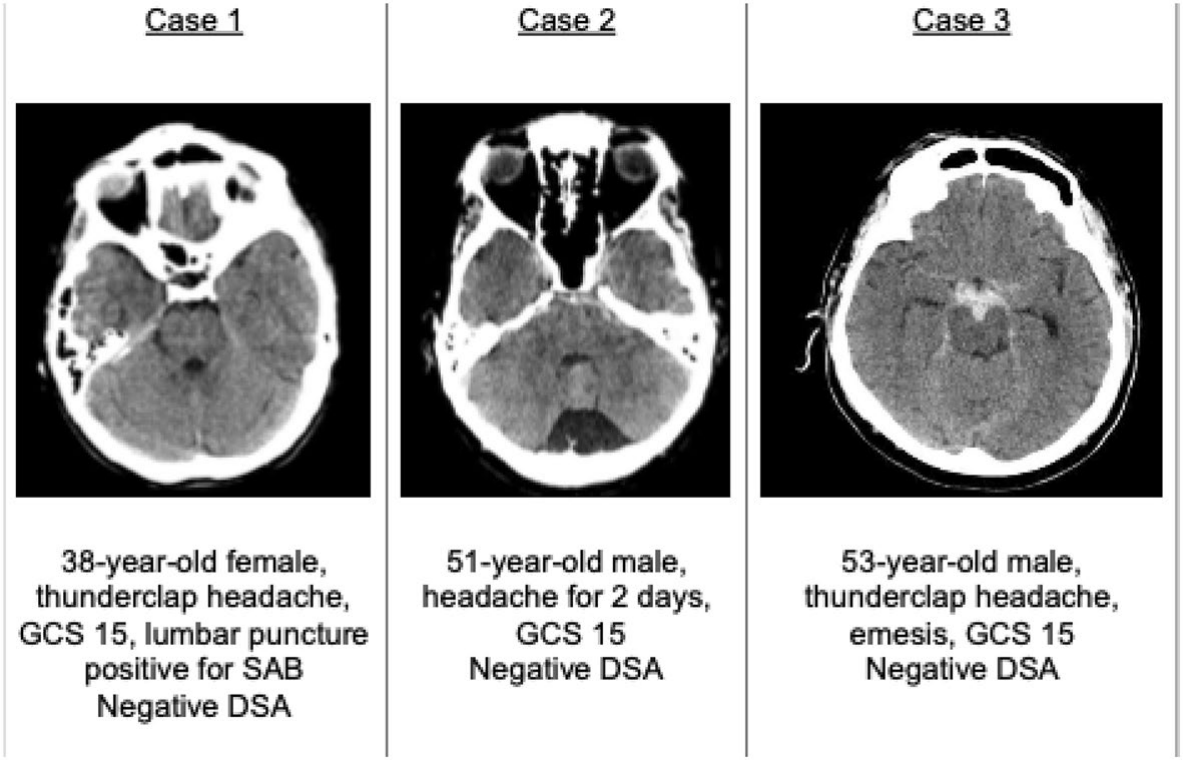
Case vignettes of minor, moderate and severe NASAH.

Additionally, three case vignettes were presented to evaluate the treatment and follow up regime in minor, moderate and severe NASAH (Fig. 1). The division in three severity scales was performed solely on the CT-scans provided and on patients’ symptoms. Validated scales as the Fisher or Hunt and Hess score were not provided, but could be applied by the centers using the information given.

### Outcome

This survey focused on the case load, rate of CV as well as DCI and treatment regime to diminish complications of patients being admitted with NASAH to neurosurgical departments in Germany.

### Statistical analysis

For categorial data, absolute numbers and percentages are given. Pearson-Chi^2^ test without Yates correction was used to map correlation. Further, contingency coefficient is applied. Reported p-values are two-sided and, where considered statistically significant, at p < 0.05. Data analysis were performed with IBM SPSS v. 25 (IBM Corp, Armonk, USA).

### Ethical approval and consent to participate

The survey was conducted in accordance with the ethical standards of the institutional and national research committee and with the 1964 Helsinki Declaration and its later amendments. The Human Investigation Committee of Ludwig-Maximilians-University Munich, Germany approved this study (Reference Nr: 21–1297). Individual consent to participate was not necessary, since the survey was conducted without real information about the patients.

## Results

**Figure 2:**
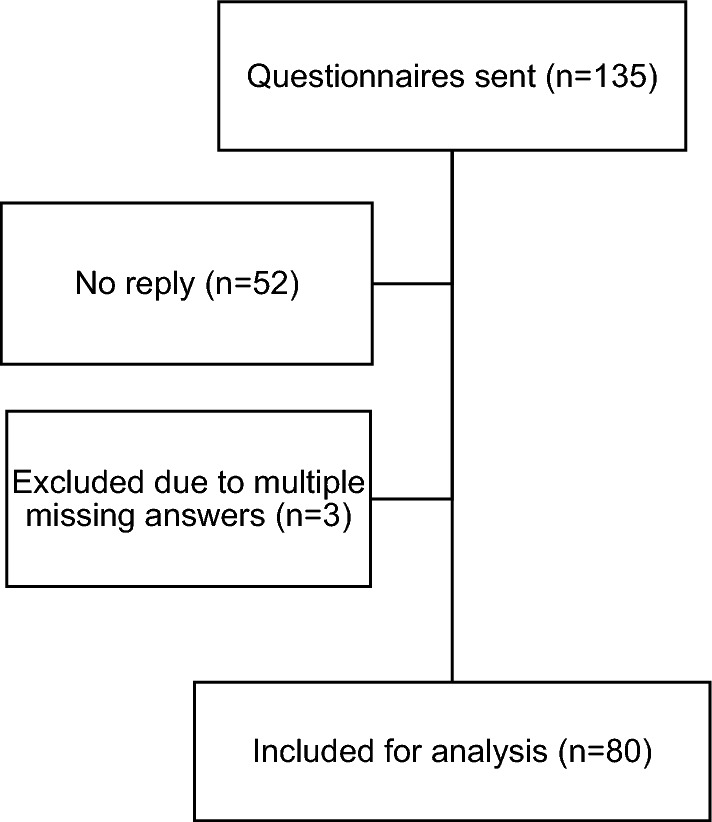
STROBE dataset of included questionnaires.

A total of 135 paper-based questionnaires were send to all neurosurgical departments in Germany. A total of 83 (61.5%) were answered, whereof three (2.2%) could not be included for evaluation due to multiple missing answers (Fig. 2). The answers were provided by neurosurgeons only.

### Case load

**Figure 3:**
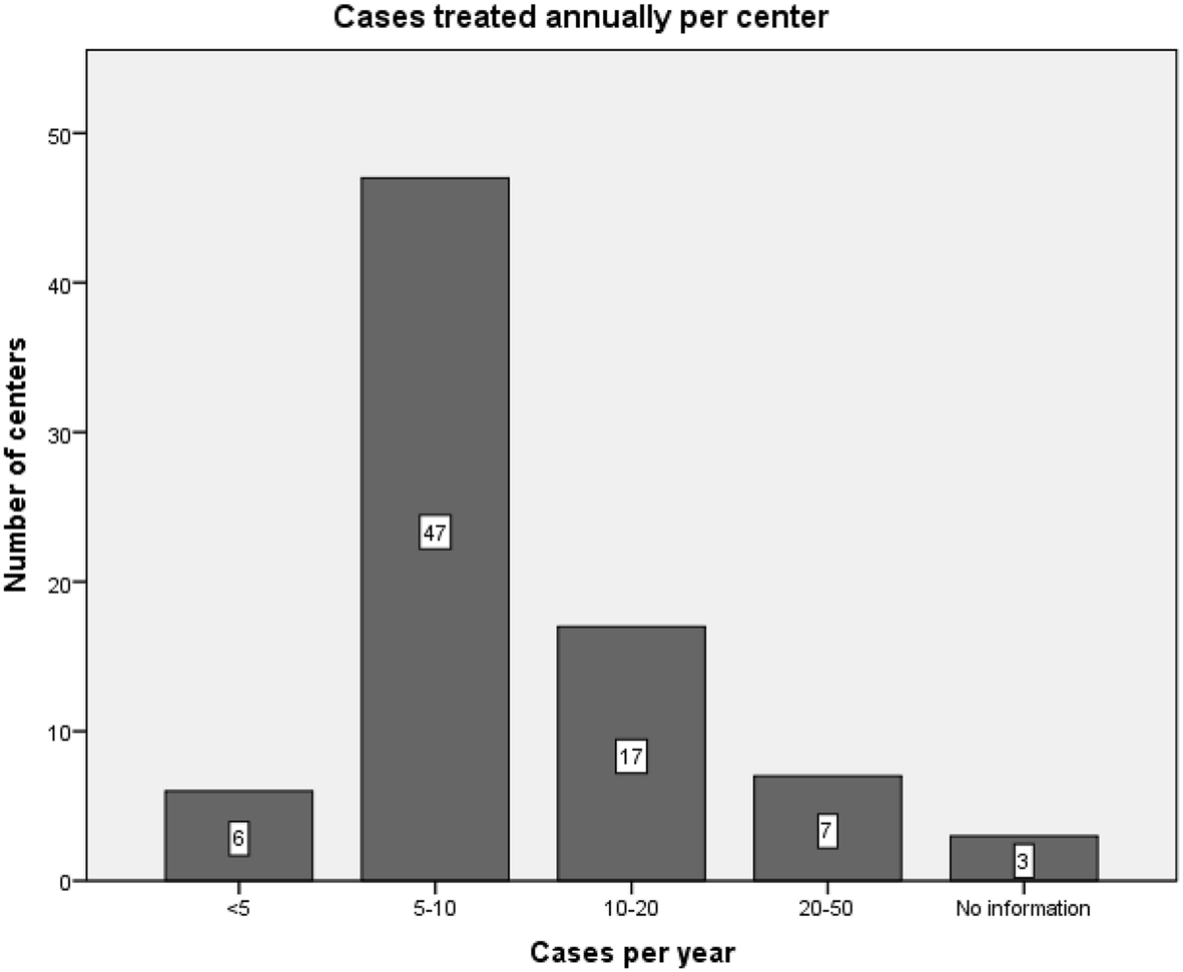
Case load per neurosurgical department.

Most centers (n = 53; 66.3%) treat less than 10 NASAH patients per year, while a case load exceeding 20 cases annually is rare (n = 7; 8.8%). A detailed view is given in Fig. 3.

### Rate of CV or CV related complications

Centers were asked to evaluate the rate of CV or CV related complications with the need for further treatment. Most centers (n = 61; 76.3%) estimate the percentage of patients suffering from CV or CV related complications from 0 and 5%.

**Figure 4:**
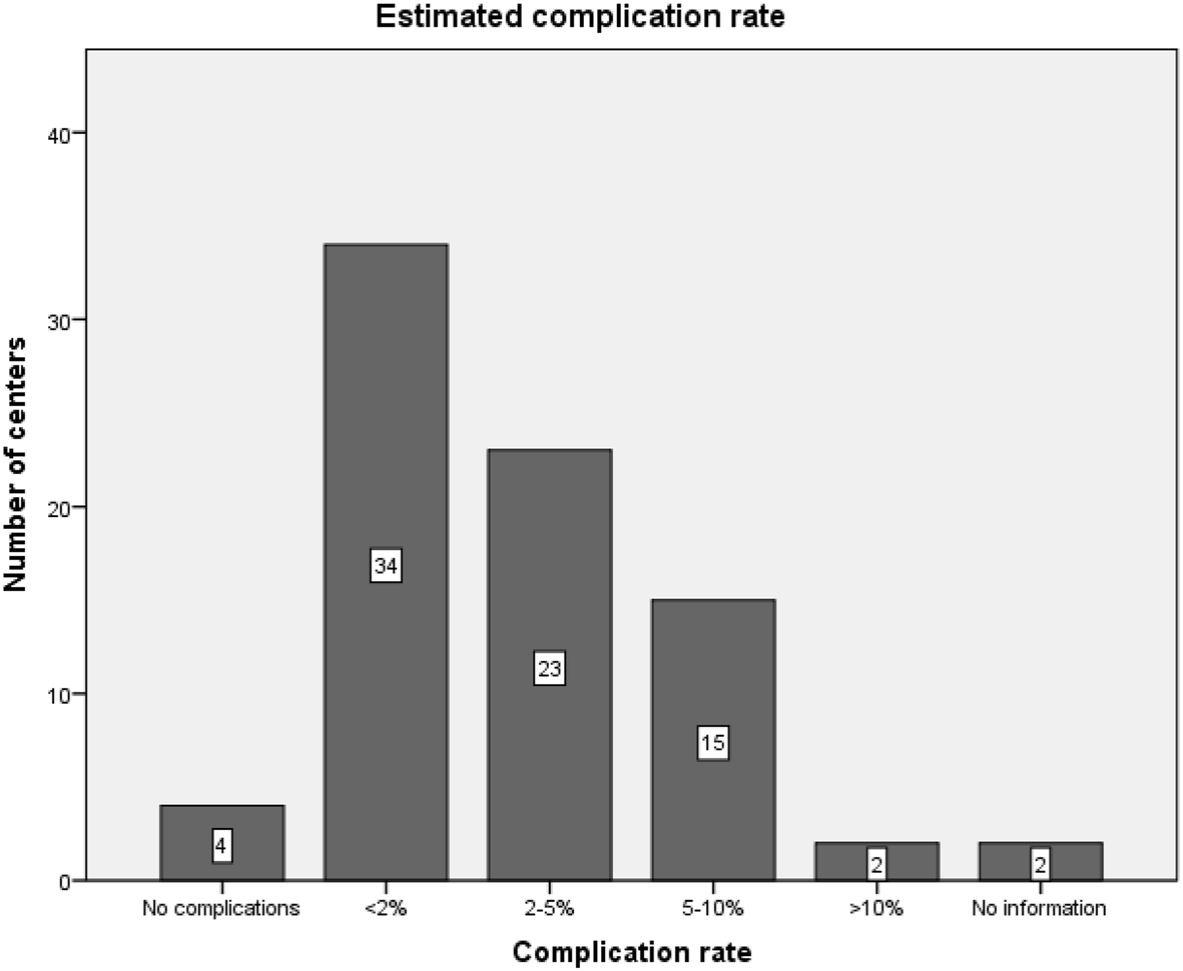
CV or CV related complications with the need for further treatment.

In two centers (2.5%) the complications are estimated with a rate exceeding 10% (Fig. 4). However, centers with a higher case load state an elevated complication rate (Chi^2^ < 0.001).

### Initial observation

In minor NASAH, 41 (51.3%) centers observe their patients on the intensive care unit (ICU), 29 (36.3%) on the intermediate care unit (IMCU) and 4 (5.0%) on the regular ward.

In moderate NASAH, 38 (47.5%) centers admit their patients to the ICU, 30 (37.5%) to the IMCU and 6 (7.5%) to the regular ward, while in severe bleeding 56 (70.0%) centers transfer their patients to the ICU, 17 (21.3%) to the IMCU and 1 (1.3%) to the regular ward.

In the remaining centers, a division into ICU or IMCU management is not possible as they operate only one of the two stations. Management of initial observation is highly dependent on the amount of perimesencephalic blood in the initial computed tomography scan (CT scan) and the clinical presentation of the patient. With statistically more patients observed on the ICU or IMCU comparing minor/moderate and severe bleeding (ICU: p = 0.02; p = 0.006; IMCU: p = 0.003; p = 0.04).

### Blood pressure management

In minor NASAH 42 (52.5%) centers perform invasive BP monitoring, while in 33 (41.3%) centers non-invasive BP monitoring seems sufficient. However, 4 (5.0%) report that continuous BP monitoring is not performed.

When comparing minor with moderate NASAH, the BP management is not statistically significantly different. In severe NASAH BP is monitored invasively in 57 (71.3%), non-invasive in 20 (25.0%) and not at all in 2 (2.5%) centers. In severe NASAH statistically significantly more centers observe their patients with invasive (p = 0.02) or non-invasive (p = 0.04) BP monitoring in comparison to minor NASAH.

### Treatment regime

Based on our survey, it was found that many departments are treating NASAH according to the protocol used for aneurysmal SAH treatment. In minor NASAH 33 centers (41.3%) perform repetitive TCD and CV prophylaxis for 14 days. Solely 5 (6.3%) perform CV prophylaxis for 14 days without TCD, whereas in 24 centers (30.0%) repeated TCD is performed without CV prophylaxis. Neither CV prophylaxis nor TCD are performed in 18 centers (22.5%).

Compared to moderate NASAH, no statistically significant change can be detected. However, in severe NASAH, more departments (n = 51; 63.8%) watch their patients carefully with TCD and CV prophylaxis for 14 days. Neurosurgical departments in Germany treat patients with severe NASAH statistically significantly more often according to the protocol used for aneurysmal SAH with repeated TCD and CV prophylaxis for 14 days (minor/severe p = 0.007; moderate/severe p = 0.03).

### Follow-up imaging

In minor NASAH most centers perform a second DSA within the hospitalization (n = 34; 42.5%). Seventeen (21.3%) perform a second DSA after 6–12 weeks, whereas the remaining 2 (2.5%) centers opt for a DSA 6–12 month after the initial bleeding. However, 27 centers (33.8%) state that they do not perform a second DSA in these patients.

Regarding moderate NASAH, 35 centers (43.8%) perform a control DSA during hospitalization and 19 (23.8%) within the first 6–12 weeks. However, solely three centers (3.8%) perform the DSA 6–12 month after ictus and 22 (27.5%) do not opt for a second DSA after a negative one. One center (1.3%) did not provide an answer.

In severe NASAH, more than half of the centers perform a second DSA during the patient’s hospital stay (n = 47; 58.8%). However, 18 (22.5%) control their patients with a second DSA after 6–12 weeks and only 4 centers (5.0%) perform the DSA 6–12 month after ictus. The remaining 11 (13.8%) do not opt for a second DSA even in cases of severe NASAH.

A third DSA is indicated from the minority of centers (minor and moderate 3.8%, severe NASAH 13.8%). In minor and severe bleeding Person Chi^2^ test confirmed positive correlation of the case load provided and the rate of a third DSA (minor Chi^2^ < 0.001, contingency coefficient = 0.72; severe: Chi^2^ < 0.001, contingency coefficient = 0.73).

Baseline data is summarized in Table 1.

**Table 1:** Baseline data.

	Minor SAH (n; %)	Moderate SAH (n; %)	Severe SAH (n; %)	p-value minor/moderate	p-value minor/severe	p-value moderate/severe
Treatment regime						
ICU	41 (51.3%)	38 (47.5%)	56 (70%)	0.75	0.02*	0.006*
IMCU	29 (36.3%)	30 (37.5%)	17 (21.3%)	1.0	0.003*	0.04*
Regular ward	4 (5.0%)	6 (7.5%)	1 (1.3%)	0.75	0.37	0.12
BP monitoring						
Invasive BP monitoring	42 (52.5%)	44 (55.0%)	57 (71.3%)	0.87	0.02*	0.048*
Non-invasive BP monitoring	33 (41.3%)	30 (37.5%)	20 (25.0%)	0.75	0.04*	0.12
No BP monitoring	4 (5.0%)	4 (5.0%)	2 (2.5%)	1.0	0.68	0.68
CV prophylaxis						
CV prophylaxis + TCD	33 (41.3%)	36 (45.0%)	51 (63.8%)	0.75	0.007*	0.03*
CV prophylaxis without TCD	5 (6.3%)	5 (6.3%)	7 (8.8%)	1.0	0.77	0.77
No CV prophylaxis but TCD	24 (30.0%)	26 (32.5%)	18 (21.3%)	0.86	0.37	0.22
Neither CV prophylaxis nor TCD	18 (22.5%)	13 (16.3%)	4 (5.0%)	0.42	0.002*	0.04*
Second DSA						
Within 3 weeks	34 (42.5%)	35 (43.8%)	47 (58.8%)	1.0	0.06	0.08
6–12 weeks	17 (21.3%)	19 (23.8%)	18 (22.5%)	0.85	1.0	1.0
6–12 months	2 (2.5%)	3 (3.8%)	4 (5.0%)	1.0	0.07	1.0
No second DSA	27 (33.8%)	22 (27.5%)	11 (13.8%)	0.49	0.005*	0.049*
Third DSA						
Third DSA	3 (3.8%)	3 (3.8%)	11 (13.8%)	1.0	0.05	0.05
No third DSA	53 (66.3%)	55 (68.8%)	55 (68.8)	0.86	0.86	1.0

*Denotes statistical significance.

## Discussion

The findings of our survey state that NASAH is a rarely treated neurosurgical disease with 66.3% of neurosurgical departments treating less than 10 patients per year. 61 centers (76.3%) estimate the rate of CV or CV associated complications with the need for further treatment ranging from 0 to 5%. Further, this survey confirms that the treatment regime differs from center to center and is individually adapted according to the initial severity of NASAH and the clinical presentation of every patient.

Most centers state low complication rates of NASAH, however, the complication rates in literature are widely different with some authors reporting complication rates of up to 48.3%, including CV in about 1.2–4%^6–8^. Setting the focus on CV or CV associated complications with the need for further treatment—the estimated rate of these complications, varying from 0 to 5% is comparable to the data given in the literature. Nevertheless, our single center study comparing perimesencephalic and non perimesencephalic SAH detected CV in 8.5% and DCI in 2.8% of the perimesencephalic SAH patients^9^. This may indicate, that CV and CV associated complications are underestimated in neurosurgical departments throughout Germany.

With the procedural risk of 0.3% of intra-arterial DSA a current guideline does not recommend repeated DSA in NASAH^1,10^. This finding was also confirmed in 2006 from Huttner and co-workers, who examined 69 patients with perimesencephalic SAH and concluded that one DSA should be performed prior to discharge to exclude a vascular bleeding source as well as CV. Including mostly Hunt and Hess Grade I and II patients, they stated that repeated DSA is likely to become obsolete in these patients^5^. Bashir and coworkers concluded, that repeated DSA may be obsolete in perimesencephalic bleeding pattern when hematoma and CV are absent in the first DSA^11^. Being further confirmed by the retrospective evaluation of Nützel et al., published in 2023^12^. However, the neurosurgeons answering our survey opted in about 70% in minor and moderate NASAH for a second DSA. This confirms that in clinical practice, the detection of an underlying vascular malformation takes precedence over the procedural risk of DSA. This is replicated in a single center study from Qatar, solely including patients with two negative DSA^13^.

This study is one of the few providing information about the in-hospital treatment, confirming that every patient received CV prophylaxis with a calcium channel blocker from the day of admission up to the second DSA 7–10 days after symptom onset^13^. However, neither blood pressure management nor the observational regime were evaluated. In the European guideline the prophylactic application of nimodipine is not recommended due to the scarce data of delayed cerebral ischemia (DCI) in NASAH^1^. Thus, our survey confirms that in severe NASAH, a statistically significant number of centers are implementing CV prophylaxis and repeated TCD.

Comparing the results of our survey to the current literature highlights the need for a separate guideline for treatment and follow-up of patients with NASAH.

### Strengths and limitations

The survey was only performed nation-wide, so conclusions about the management of NASAH in other countries may not be eligible. Even though the response rate was high, not all centers answered the survey and therefore the conclusions may be biased. In addition, the number of NASAH patients per year and complication rates cannot be objectively reviewed. Because our survey mainly focused on CV and CV related complications as well as DSA, other complications and diagnostics are untended.

## Conclusion

This study confirms that neurosurgical departments throughout Germany treat NASAH differently and that the initial blood distribution detected in the CT scan does influence the clinical treatment. Therefore, we suggest to conceive new treatment guidelines including this finding.

### Supplementary Information

Supplementary Information.

## Data Availability

The data that support the findings of this study are not openly available due to reasons of sensitivity and are available from the corresponding author upon reasonable request.
