# Supplementary material for: Management of perimesencephalic nonaneurysmal subarachnoid hemorrhage: a national survey
Source: Sci Rep. 2023 Aug 7;13:12805. doi: 10.1038/s41598-023-39195-2 (PMC10406943; doi:10.1038/s41598-023-39195-2)
Supplement: Supplementary file 1 — Supplementary Information. [file 41598_2023_39195_MOESM1_ESM.pdf]

Survey non-aneurysmatic, non-traumatic SAH

A) How many cases of non-aneurysmal / non-traumatic SAH are treated in your hospital per year?

a) <5
b) 5-10
c) 10-20
d) 20-50
e) No information

B) In how many cases do you diagnose cerebral vasospasm / vasospasm related complications with the need for further treatment?

a) <2%
b) 2-5%
c) 5-10%
d) >10%
e) We never diagnosed patients with non-aneurysmal / non-traumatic SAH with vasospasm or related complications

	Case 1	Case 2	Case 3
	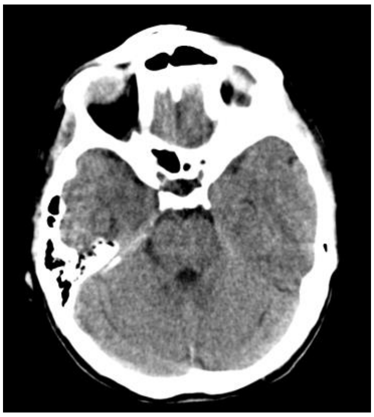 38-year-old female, thunderclap headache, GCS 15, lumbar puncture positive for SAB Negative DSA	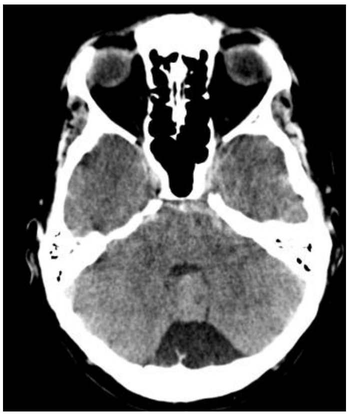 51-year-old male, headache for 2 days, GCS 15 Negative DSA	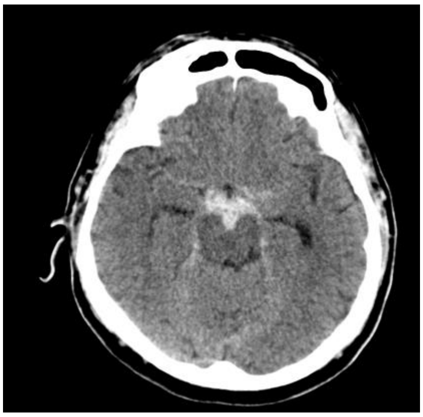 53-year-old male, thunderclap headache, emesis, GCS 15 Negative DSA
1			
2			
3			
4			
5			

1. What is your treatment regime?

a) Transfer to the Intensive care unit
b) Transfer to intermediate care station
c) Transfer to regular ward

2. What is your treatment regime?

a) blood pressure monitoring (invasive)
b) blood pressure monitoring (non-invasive)
c) No blood pressure monitoring

3. What is your treatment regime?

a) cerebral vasospasm prophylaxis for 14 days with repetetive transcranial ultrasound
b) cerebral vasospasm prophylaxis for 14 days without repetetive transcranial ultrasound
c) No cerebral vasospasm prophylaxis but with repetetive transcranial ultrasound
d) No cerebral vasospasm prophylaxis and without repetetive transcranial ultrasound

4. When do you perform a second angiography in this case?

a) During hospital stay within the first 3 weeks after ictus
b) 6-12 weeks after ictus
c) 6 to 12 months after ictus
d) We do not perform another DSA in this patient

5. If the 2nd DSA is negative, do you opt for a third angiography in this case?

a) Yes
b) No
